# Quantitative Evaluation by Digital Pathology of Immunohistochemical Expression of CK7, CK19, and EpCAM in Advanced Stages of NASH

**DOI:** 10.3390/biomedicines12020440

**Published:** 2024-02-16

**Authors:** Daniela Cabibi, Antonino Giulio Giannone, Alberto Quattrocchi, Vincenza Calvaruso, Rossana Porcasi, Domenico Di Grusa, Anna Maria Pavone, Albert Comelli, Salvatore Petta

**Affiliations:** 1 Unit of Anatomic Pathology, Department of Health Promotion Mother and Child Care Internal Medicine and Medical Specialties (PROMISE), University Hospital AOU Policlinico “P. Giaccone”, University of Palermo, Via del Vespro 129, 90127 Palermo, Italy; cabibidaniela@virgilio.it (D.C.); alberto.quattrocchi@unipa.it (A.Q.); r.porcasi@libero.it (R.P.);; 2 Section of Gastroenterology and Hepatology, Department of Health Promotion Mother and Child Care Internal Medicine and Medical Specialties (PROMISE), University Hospital AOU Policlinico “P. Giaccone”, University of Palermo, Via del Vespro 129, 90127 Palermo, Italy; 3 Ri.MED Foundation, Via Bandiera 11, 90133 Palermo, Italy; ampavone@fondazionerimed.com (A.M.P.); acomelli@fondazionerimed.com (A.C.)

**Keywords:** NASH, QuPath, digital pathology, liver biopsy, immunohistochemistry, biliary metaplasia, ductular proliferation

## Abstract

(1) Background: Nonalcoholic Steatohepatitis/Nonalcoholic Fatty Liver Disease (NASH/NAFLD) is the most recurrent chronic liver disease. NASH could present with a cholestatic (C) or hepatic (H) pattern of damage. Recently, we observed that increased Epithelial Cell Adhesion Molecule (EpCAM) expression was the main immunohistochemical feature to distinguish C from H pattern in NASH. (2) Methods: In the present study, we used digital pathology to compare the quantitative results of digital image analysis by QuPath software (Q-results), with the semi-quantitative results of observer assessment (S-results) for cytokeratin 7 and 19, (CK7, CK19) as well as EpCAM expression. Patients were classified into H or C group on the basis of the ratio between alanine transaminase (ALT) and alkaline phosphatase (ALP) values, using the “R-ratio formula”. (3) Results: Q- and S-results showed a significant correlation for all markers (*p* < 0.05). Q-EpCAM expression was significantly higher in the C group than in the H group (*p* < 0.05). Importantly ALP, an indicator of hepatobiliary disorder, was the only biochemical parameter significantly correlated with Q-EpCAM. Instead, Q-CK7, but not Q-CK19, correlated only with γGlutamyl-Transferase (γGT). Of note, Stage 4 fibrosis correlated with Q-EpCAM, Q-CK19, and ALP but not with γGT or ALT. Conclusions: Image analysis confirms the relation between cholestatic-like pattern, associated with a worse prognosis, with increased ALP values, EpCAM positive biliary metaplasia, and advanced fibrosis. These preliminary data could be useful for the implementation of AI algorithms for the assessment of cholestatic NASH.

## 1. Introduction

Nonalcoholic Fatty Liver Disease (NAFLD) is a complex spectrum of chronic liver diseases featured by hepatic fat accumulation [1]. NAFLD is properly diagnosed when alcohol consumption or other secondary causes that lead to hepatic fat accumulation are excluded. Nonalcoholic Steatohepatitis (NASH) is the worst histological presentation of the NAFLD spectrum, and it could lead to various stages of fibrosis [2].

Although histology is not routinely required for NAFLD diagnosis, to date, liver biopsy is considered the “gold standard”, as NAFLD is often asymptomatic and many patients have normal liver function tests [3,4,5].

Increased values of alanine aminotransferase (ALT) are present in a significant proportion of patients as an expression of hepatic inflammation and cytolysis. However, cholestatic presentation with an increase in alkaline phosphatase (ALP) is also observed in several cases [6,7,8,9].

In drug-induced liver injury, these biochemical parameters had been employed in the calculation of an R-ratio formula to discriminate hepatitis-like from cholestatic-like patterns of damage [10,11].

Recently, the R-ratio formula has been applied to NASH [12,13]. In NASH, the biochemical cholestatic pattern (C pattern) is associated with advanced stages of histological fibrosis, more severe liver injury, a different metabolic pattern [12], and major liver-related outcomes [13] compared with the biochemical hepatitic pattern (H pattern).

Cytokeratins (CK) are a group of structural proteins that are part of the class constituting intermediate filaments of the cytoskeleton. CKs are diffusely present in almost all the different types of epithelial cells, and so they are used as markers to specifically identify epithelial cells. Classically, CKs are distinguished into two types: type I CKs are acidic proteins, whereas type II are basic [14,15].

Normal bile ducts and bile ductular reactions, which occur in many chronic liver diseases, express cytokeratin 7 (CK7) and cytokeratin 19 (CK19). Therefore, ductular reaction can be highlighted by immunohistochemistry for these cytokeratins [16,17]. 

Epithelial Cell Adhesion Molecule (EpCAM) is a membrane glycoprotein mediating homophylic cell–cell adhesion in simple, transitional, and pseudostratified epithelia [18,19,20,21,22]. Moreover, it is expressed in the embryonic liver, in both proliferating hepatocytes and bile ducts. However, its expression in adult liver is retained only at small bile ducts and canaliculi, whereas adult hepatocytes are EpCAM-negative [23]. Of note, it has been reported that the expression of EpCAM is reactivated during liver regeneration, marking intermediates liver progenitor cells [24,25]. However, the diagnostic and predictive significance of EpCAM in other contexts of liver disease, such as NASH, has been not considered so far.

Various types of liver bile duct injuries are featured with the proliferation of duct-like intra-hepatic structures. We previously defined biliary metaplasia as the presence of single cells or small clusters of cells without a clear central lumen, showing EpCAM and CK7 co-expression, indicative of an intermediate hepatobiliary immunophenotype (so-called intermediate hepatocytes). Conversely, ductular proliferation was defined as the presence of newly formed small ducts, located outside the portal tracts, showing CK7/CK19 co-expression, indicative of a mature biliary immunophenotype. Moreover, we reported that NAFLD patients with the C pattern were characterized by higher amounts of biliary metaplasia than patients with the H pattern, semi-quantitatively evaluated by an experienced pathologist observer [13]. Importantly, we observed that increased EpCAM expression was the most discriminating feature to distinguish the C pattern from the H pattern, with statistically significant differences between the two groups [13].

However, the semi-quantitative evaluation, although it provided statistically significant results, presents the limitation of subjectivity, as it does not allow the precise quantification of the immunohistochemical expression.

The aim of this study is to use digital pathology and the support of software image analysis for a quantitative assessment of immunohistochemical expression of CK7, CK19, and EpCAM in NASH liver biopsies with C and H patterns and with advanced stages of fibrosis, to verify the reproducibility of the results obtained from the semi-quantitative evaluation of the observer, in order to provide the first data useful for further studies for the creation of AI algorithms. Moreover, we investigated the biochemical and morphological features associated with the measured levels of these immunohistochemical markers in NASH liver biopsies.

## 2. Materials and Methods

### 2.1. Patients and Liver Biopsies

We retrospectively selected 47 patients seen by the Gastroenterology and Hepatology Unit of the University Hospital of Palermo between 2007 and 2019, who underwent needle liver biopsy and were histologically diagnosed as having NASH with fibrosis stage 3–4 according to Kleiner’s score [26]; the biochemical parameters were recovered from clinical records. Many of them (36/47) were included in the larger multicenter case series of our previously published study [13]. Based on clinical records, a history of alcohol consumption was excluded through a questionnaire, ruling out cases of >30 g/day of consumption in men and >20 g/day in women. Moreover, for each patient with elevated ALP, biliary obstruction, multifocal intrahepatic, and extrahepatic biliary strictures, as well as the presence of antimitochondrial antibodies or antinuclear antibodies, highly specific of primary biliary cirrhosis, have been excluded. In the group of 47 selected patients, 48% presented obesity with a BMI ≥ 30, 33% were affected by diabetes, and 39% by hypertension, defined as systolic blood pressure ≥ 140 mm Hg and/or diastolic blood pressure ≥ 90 mm Hg or use of blood pressure-lowering agents. The mean value ± standard deviation of total cholesterol was 192 mg/dL ± 43.

### 2.2. R-Ratio Calculation

According to the ALT and ALP biochemical parameters, all 47 patients were classified into the cholestatic group (group C, with C pattern, 22 cases) or the hepatitis group (group H, with H pattern, 25 cases) based on the ratio (R), which was obtained as a result of the following formula [10,11,12]:

R = (ALT/ALT at the upper limit of normal)/(ALP/ALP at the upper limit of normal);

For ALT, the upper limit of normal was considered 19 IU/L in women and 31 IU/L in men; for ALP, the upper limit of normal was assumed to be 115 IU/L, based on the laboratory reference values.

When R < 2, the patient was included in group C; when R > 5, the patient was included in group H. In 2 cases, the R-value was between 2 and 5 and they were initially considered as a mixed group; however, since the mixed pattern was previously reported to be more similar to the H pattern [12], the cases were included in group H [Table 1].

### 2.3. Histological and Immunohistochemical Analysis

All sections from liver biopsies of the 47 patients were stained with Hematoxylin-Eosin, PAS-D, Sirius Red, and Shikata’s Orcein staining. As a histological selection criterion, only liver biopsies with a portal tract length between 1.5 and 10 cm were included in the study.

Immunohistochemical (IHC) staining was performed with the automated Ventana BenchMark Ultra staining system (Ventana/Roche Tissue Diagnostics, Tucson, AZ, USA) according to the instructions of the manufacturers, using the following pre-diluted primary antibodies: anti-cytokeratin 7 (CK7, clone SP52; rabbit monoclonal; Ventana/Roche Tissue Diagnostics, Tucson, AZ, USA), anti-cytokeratin 19 (CK19, clone A53-B/A2.26; murine monoclonal; Cell Marque, Rocklin, CA, USA), and anti-EpCAM (Ber-EP4 clone; murine monoclonal; Cell Marque, Rocklin, CA, USA).

### 2.4. Digital Image Analysis

Sections stained with immunohistochemical techniques using CK7, CK19, and EpCAM were digitalized as whole slide images (WSIs) in tiff format at 40× magnification with Aperio CS, Leica Microsystems. Image analysis was conducted with the QuPath software package (version 0.4.3).

The analysis was performed on the entire section of the needle biopsy specimen to avoid selection bias.

Using QuPath software, each image was subjected to automatic correction of the image color scales, through the software’s ‘Estimate stain vectors’ function, to perform the automatic correction of color scale. Subsequently, the areas of interest were measured in μm^2^ in the hematoxylin channel, whereas the IHC positive areas were measured in the DAB channel, both at 0.5 μm/pixel resolution.

For each case, the percentage ratio (RQuPath) was calculated using the following formula: RQuPath = Positive Area/Total Area × 100.

### 2.5. Semi-Quantitative Scoring System for Histological Analysis

An expert pathologist, unaware of results from digital analysis, analyzed the sections from liver biopsies and classified them according to the following semi-quantitative scoring criteria:

Kleiner’s score [26] was used for the histological assessment of NAFLD and specifically to grade steatosis, lobular inflammation, hepatocellular ballooning on hematoxylin-eosin stained sections, and the stage of fibrosis from 0 to 4 on Sirius red-stained sections.

The presence of ductular proliferation was defined as the presence of small, newly formed ducts, located beyond the portal tracts, with immunohistochemistry positivity for CK7 and CK19.

Similarly, biliary metaplasia was defined as the presence of single cells or small clusters of cells without a defined central lumen, with positive expression for CK7 and EpCAM, suggestive of an intermediate hepatobiliary phenotype.

According to the above-mentioned markers, ductular proliferation and biliary metaplasia were semi-quantitatively scored as follows: score 0 (absence outside the portal tracts); score 1 (focal presence close to the portal tracts); score 2 (moderate presence adjacent to the portal tracts in less than 50% of the portal tracts, in the range of 1 High Power Field (HPF) from the portal tract); and score 3 (widespread presence of immunohistochemical expression adjacent in more than 50% of the portal tracts, in the range of more than 1 HPF from the portal tract).

### 2.6. Statistical Analysis

Spearman’s correlation coefficient, point-biserial correlation, Student’s *t*-test, and the Wilcoxon test were performed by using SPSS software v.21 (IBM). To perform point-biserial correlation, we transformed the ordinal variables of the semi-quantitative scoring into dichotomous variables of positive or negative results as follows: for ductular proliferation and biliary metaplasia, the values 0–1 = 0 and the values 2–3 = 1; for fibrosis the value 3 = 0, the value 4 = 1. All *p* values < 0.05 were considered statistically significant.

## 3. Results

Digital and semi-quantitative analyses were conducted on liver biopsies stained with immunohistochemical markers of biliary structures CK7, CK19, and EpCAM. The prefix “Q” indicates the quantitative results obtained with QuPath digital analysis, whereas the prefix “S” refers to the semi-quantitative results obtained by the observer. We first verified the correlation between the two analyses (Table 2). Spearman’s Rho test showed a significant statistical correlation between S- and Q-results for all three tested markers (*p* < 0.01 for CK7 and EpCAM expression; *p* < 0.05 for CK19 expression).

Next, we tested if Q-results were informative of patients’ group classification and histological evaluation.

**Figure 1:**
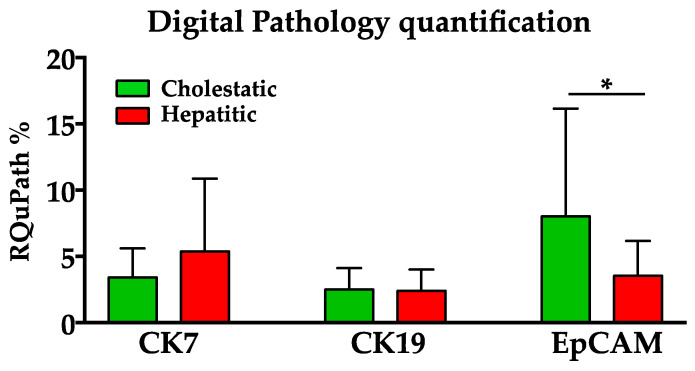
Quantification of CK7, CK19, and EpCAM markers on immunohistochemistry staining of liver biopsies. Q-EpCam expression was significantly higher in the cholestatic group than in the hepatitic group, while no significant statistical difference was found for Q-CK19 and Q-CK7 between the two groups. * = *p* < 0.05.

**Figure 2:**
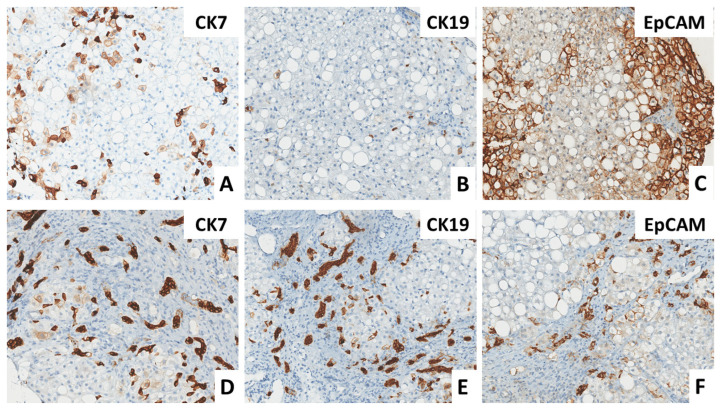
(**A**–**C**) Patient with a cholestatic pattern, characterized by biliary metaplasia of hepatocytes, with increased expression of CK7 (**A**), very rare CK19 (**B**) positive cells, and diffuse positivity for EpCAM (**C**). (**D**–**F**) patient with a hepatitic biochemical pattern, characterized by ductular reaction with proliferation of small ductules positive for CK7 (**D**) and CK19 (**E**) and a slight increase in EpCAM expression (**F**). Immunoperoxidase stain. Original magnification 200×.

Q-EpCam expression was slightly higher in group C (mean EpCAM RQuPath = 8.0%) than in group H (mean EpCAM RQupath = 3.5%). This difference, although not impressive, resulted in statistical significance (*t*-test: *p* < 0.05; Wilcoxon’s test: *p* < 0.05). By contrast, no significant statistical difference was found for Q-CK7 and Q-CK19 between the two groups (Figure 1). As expected, CK7 was strongly correlated with both markers: EpCAM (Spearman’s Rho= 0.586, *p* < 0.01) and CK19 (Spearman’s Rho = 0.726; *p* < 0.01) (Figure 1 and Figure 2).

To define the clinical features related to the markers measured with digital pathology, we performed a correlation analysis combining measured values of Q-CK7, Q-K19, and EpCAM with fibrosis and biochemical parameters (Table 2). All Q-makers positively correlated with fibrosis, with a more significant correlation for Q-CK7 and Q-CK19. We found that Q-CK19 did not correlate with γGT, ALP, or ALT. Q-CK7 correlated only with γGT; Q-EpCAM significantly correlated only with ALP, but not with ALT nor with γGT values. Considering the biochemical parameters, ALP correlated with EpCAM, with pattern C of NASH, and with stage 4 of fibrosis. Surprisingly, ALT did not correlate with any morphological features.

Noteworthily, the correlation of both Q-EpCAM and ALP values with stage 4 fibrosis further supported that Q-EpCAM values are overall related to the cholestatic presentation of NAFLD (Table 3).

The γGT values were related to ALT and ALP values but not to an advanced stage of fibrosis. ALT values correlated with the H pattern, and γGT values, but not with any immunohistochemical marker, nor with stage 4 of fibrosis (Table 2). Strikingly, ALP was the only biochemical parameter correlating with an advanced stage of fibrosis in NASH, while EpCAM was the only immunohistochemical marker showing a correlation with ALP.

## 4. Discussion

To date, the histological assessment of liver biopsies performed by an experienced pathologist is considered the gold standard for NASH diagnosis. The need to implement and validate the use of new tools has emerged with the progress of digital pathology, image analysis, and AI. Recently in the literature, many studies have appeared concerning the utility of these tools in the histological diagnosis of liver diseases, including NASH [27,28].

Our study aimed to compare the results of the semiquantitative assessment (S-results) of histological and immunohistochemical features of NASH obtained by observation of the pathologist [13] with the results of the quantitative assessment of digital analysis by using QuPath (Q-results).

Furthermore, in order to understand the clinical meaning of Q- and S-results, the relation with the biochemical parameters was investigated.

Q-results about CK7, CK19, and EpCAM expression were in line with the S-results of our previous study [13], proving that digital image analysis could be considered a useful tool to quantify the expression of these immunohistochemical markers.

In detail, Q-analysis confirmed the absence of significant differences between group C and group H regarding CK7 and CK19 expression, which were similarly detected in both groups. CK7 is mainly expressed in association with EpCAM in biliary metaplasia and with CK19 in ductular proliferation. On the contrary, Q-EpCAM was more expressed in group C than in group H, with a statistically significant difference. Therefore, Q-EpCAM was revealed to be a reliable immunohistochemical marker to distinguish the two groups (Figure 1 and Figure 2). Moreover, it was the only marker related to ALP that, in turn, showed to be the only biochemical parameter related to stage 4 of fibrosis (Table 2).

This study underlines the relation between cholestatic C pattern with increased ALP values, EpCAM positive biliary metaplasia, and a more advanced stage of fibrosis.

Recent studies in cirrhotic and non-cirrhotic patients with NAFLD report the association between the C pattern and portal hypertension [29] and, in keeping with our observations, underline the importance of recognizing the C pattern.

**Figure 3:**
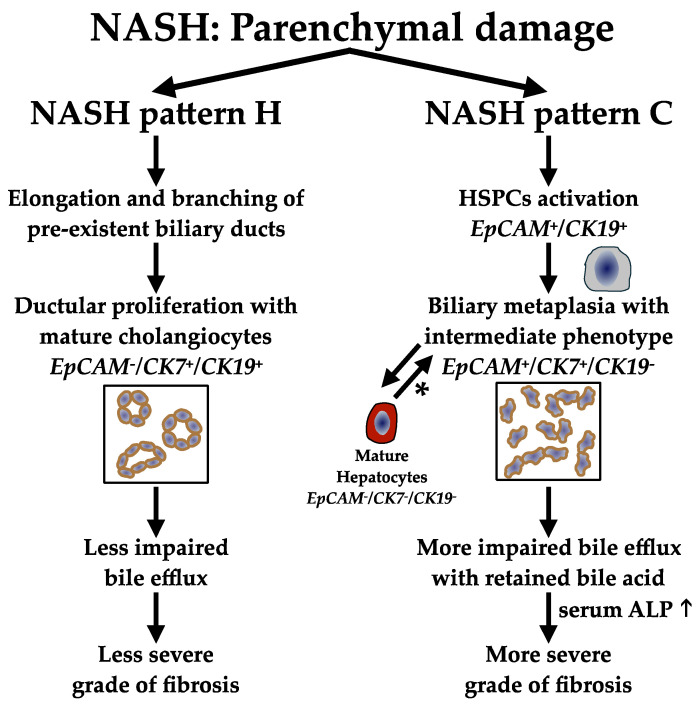
Pathways of NASH parenchymal damage associated with H and C patterns. The left scheme presents the route more typically associated with a hepatitic histologic pattern of NASH, with the formation of more mature ductular structures, expressing both CK7 and CK19. These types of structures, which are more functional, are more efficient in allowing bile efflux, ultimately leading to a less severe grade of fibrosis. Conversely, in cholestatic patterns, it is likely that immature ducts, still expressing EpCAM other than CK7, but almost negative for CK19, determine higher retention of bile acids, leading to serum ALP increase and a more advanced grade of fibrosis, as shown in the right scheme. Note that the two pathways are not mutually exclusive, and the general balance between the two routes, together with other factors, determines the ultimate outcome of fibrosis. * According to the double differentiation/de-differentiation histogenetic pathways [30]. H, hepatitic; C, cholestatic; HSPCs, hepatic stem/progenitor cells; ALP, alkaline phosphatase; EpCAM, Epithelial Cell Adhesion Molecule; CK7, cytokeratin 7; CK19, cytokeratin 19.

Our study suggests that in NASH with cholestatic pattern and stage 4 of fibrosis, where inflammatory phenomena are often mild, the toxic effect of intrahepatic retention of bile acids could play a more effective fibrogenic role than cytolytic phenomena. In Figure 3, we illustrate the hypothetic pathways leading to the different ductular reactions of C and H patterns of NASH, as well as their effects on fibrosis.

It has been previously reported that bile acid concentration and ALP are correlated.

Particularly, a reduction of bile acid canalicular secretion, together with secondary bile acid retention, leads to a liver ALP synthesis increase [31]. Desmet hypothesized that bile acid overload may be an early trigger in biliary metaplasia or dedifferentiation of hepatocytes, exerting a stimulus on parenchymal cells. The latter in turn activates the hepatic stellate cells (HSCs) that proliferate with a fibrogenic effect [30,32,33].

Chronic liver disorders of different etiopathogenesis can activate hepatic progenitor cells (HPCs), normally maintaining a quiescent state, and this activation results in a ductular reaction. HPCs can differentiate into intermediate cells and subsequently into hepatocytes or cholangiocytes. Moreover, hepatocytes can de-differentiate towards an EpCAM+ “intermediate” phenotype [24,34,35].

Other authors stated that the activation of HPCs, more than their specific phenotype, could play a significant role in the progression to cirrhosis and a more aggressive course of the disease [36].

The biological importance of HPC activation has also been investigated in NAFLD. Previously we hypothesized that in the cholestatic type of NASH, intrinsic biliary dysfunction leads to the presence of biliary metaplasia, a reaction induced by bile acid overload and not fully compensated by effective ductular differentiation. This process could exacerbate the biochemical and histological features of cholestasis and, by activating HPCs, could trigger fibrogenic pathways, with an increased risk of progression [13].

Noteworthily, we found a correlation between biochemical cholestasis, indicated by the increase of ALP values, and the EpCAM expression with the presence of immature “intermediate hepatocytes”.

In our study, γGT value results were related to ALT and ALP values but not to EpCAM expression, pattern C, and advanced stage of fibrosis (Table 2).

γGT is a transmembrane protein expressed on the cell membrane, primarily on biliary epithelial cells. It has high diagnostic sensitivity for cholestasis but low specificity, as it can be related to several diseases (e.g., diabetes, obesity, alcoholism) and a wide variety of drugs [37], and sometimes it is abnormal in patients with no primary hepatobiliary disease [38]. Moreover, it has to be considered that the increases in serum γGT values could also be determined by drug-induced biliary hyperplasia or by increased pressure of the biliary system due to structural cholestasis, other than enzyme release following damage of biliary epithelial cells [39]. In keeping with these statements, in our study, γGT showed a relation with ALT values. Of note, no correlation was found with pattern C of NASH nor with advanced fibrosis, suggesting that, at least in some patients, the increase of γGT could be not related to toxic biliary acids retention with fibrogenic effects. NASH patients in fact are often obese, diabetic, and take several drugs, and γGT increase could be related to these co-morbidities.

Thus, ALP is the more specific index of intra-hepatic cholestasis, and, in our study, it was the only biochemical parameter correlating with the advanced stage of fibrosis, which could explain the worse prognosis reported in patients with cholestatic patterns of NASH. Q-EpCAM was the only immunohistochemical marker showing a correlation with ALP, a marker of cholestasis, and was related to a potentially worse prognosis since both EpCAM and ALP independently correlated either with fibrosis stage 4. Of note, γGT and ALT values were not correlated with fibrosis stage 4 (Table 2).

In conclusion, we confirm through image analysis the existence of NASH with a cholestatic pattern, which is an important result since previous studies have already shown a worse prognosis in this group compared to NASH patients with a hepatitic pattern.

To the best of our knowledge, the quantitative expression of EpCAM, evaluated using digital pathology and its correlation with the different patterns of NASH, has never been studied. These results are preliminary and limited by the restricted number of cases. However, if confirmed on a larger casuistry and validated by multicentric studies, they could be exploited to develop algorithms based on artificial intelligence, allowing in the future a faster and more objective evaluation of cholestatic NASH cases and their prognostic significance.

Moreover, previous studies hypothesized that “NAFLD and cholestatic diseases share key pathophysiological mechanisms that may be targeted by novel therapeutic concepts” [40]. In line with these observations, the analytical quantitative studies could be extended to chronic cholestatic diseases such as primary biliary cholangitis and primary sclerosing cholangitis, in order to evaluate the role of EpCAM and its relationship with ALP, fibrosis, and prognosis of the patients.

Finally, the present study might suggest the potential role of therapy with anticholestatic drugs in NASH [41,42], paving the way for a more effective treatment of these patients.

## Figures and Tables

**Table 1:** General and clinical information of the studied patients.

	N	Gender	Age		ALP (U/I)		γGT (U/I)		ALT (U/I)	
		M/F%	Median	IQR	Median	IQR	Median	IQR	Median	IQR
C Pattern	22	62/38	63	54–65	94	75–108	59	43–97	33	29–50
H Pattern	25	48/52	58	51.5–62	62	55.5–80.5	76	51.5–154	91	70–128.5
Total	47	53/47	58.5	56–98	67.50	56–98	76	48–113	70	42–119.75

**Table 2:** Bivariate correlation of Spearman between semiquantitative (S) and quantitative (Q) assessment of immunohistochemical markers ^a^.

	S-CK7	S-CK19	S-EpCAM	Q-CK7	Q-CK19	Q-EpCAM
S-CK7	1.000	0.737** 0.000	0.561 ** 0.001	0.468 ** 0.006	0.496 ** 0.003	0.453 ** 0.008
S-CK19	0.737 ** 0.000	1.000	0.369 * 0.029	0.433 ** 0.009	0.420 * 0.012	0.382 * 0.024
S-EpCAM	0.561 ** 0.001	0.369 * 0.029	1.000	0.597 ** 0.000	0.533 ** 0.001	0.816 ** 0.000
Q-CK7	0.468 ** 0.006	0.433 ** 0.009	0.597 ** 0.000	1.000	0.726 ** 0.000	0.586 ** 0.000
Q-CK19	0.496 ** 0.003	0.420 * 0.012	0.533 ** 0.001	0.726 ** 0.000	1.000	0.744 ** 0.000
Q-EpCAM	0.453 ** 0.008	0.382 * 0.024	0.816 ** 0.000	0.586 ** 0.000	0.744 ** 0.000	1.000

^a^ Per each cell, the first value indicates the correlation coefficient (Spearman’s Rho), and the second is the relative *p* value (2-tailed significance). ** = *p* < 0.01 (dark green), * = *p* < 0.05 (light green).

**Table 3:** Bivariate correlation of Spearman between biochemical parameters and quantitative assessment of immunohistochemical markers ^a^.

	Q-CK7	Q-CK19	Q-EpCAM	Pattern C ^b^	Fibrosis Stage 4	ALP	γGT	ALT
Fibrosis stage 4	0.620 **	0.538 **	0.417 *	0.112	1.000	0.329 *	0.313	−0.036
	0.000	0.001	0.011	0.515		0.050	0.063	0.836
ALP	0.218	0.230	0.387 *	0.379 *	0.329 *	1.000	0.391 *	0.006
	0.201	0.177	0.020	0.023	0.050	.	0.018	0.971
γGT	0.347 *	0.103	−0.013	−0.251	0.313	0.391 *	1.000	0.394 *
	0.038	0.549	0.938	0.140	0.063	0.018	.	0.017
ALT	0.094	0.007	−0.070	−0.724 **	−0.036	0.006	0.394 *	1.000
	0.585	0.970	0.684	0.000	0.836	0.971	0.017	

^a^ Per each cell, the first value indicates the correlation coefficient (Spearman’s Rho), and the second is the relative *p* value (2-tailed significance). ** = *p* < 0.01 (dark green), * = *p* < 0.05 (light green). ^b^ C and H correlations are indicated respectively as positive and negative values. ALP, alkaline phosphatase; γGT, Gamma-glutamyl transferase; ALT, alanine transaminase.

## Data Availability

The data presented in this study are available on reasonable request from the corresponding author.

## Abbreviations

ALP, alkaline phosphatase; ALT, alanine transaminase; CK7/CK19, cytokeratins 7/19; EpCAM, Epithelial Cell Adhesion Molecule; HPF, High Power Field; NAFLD, Nonalcoholic Fatty Liver Disease; NASH, Nonalcoholic Steatohepatitis; γGT, γGlutamyl-Transferase.

## References

[1] Farrell G.C., Larter C.Z. (2006). Nonalcoholic fatty liver disease: From steatosis to cirrhosis. Hepatology.

[2] Taylor R.S., Taylor R.J., Bayliss S., Hagström H., Nasr P., Schattenberg J.M., Ishigami M., Toyoda H., Wai-Sun Wong V., Peleg N. (2020). Association Between Fibrosis Stage and Outcomes of Patients With Nonalcoholic Fatty Liver Disease: A Systematic Review and Meta-Analysis. Gastroenterology.

[3] Portillo-Sanchez P., Bril F., Maximos M., Lomonaco R., Biernacki D., Orsak B., Subbarayan S., Webb A., Hecht J., Cusi K. (2015). High Prevalence of Nonalcoholic Fatty Liver Disease in Patients With Type 2 Diabetes Mellitus and Normal Plasma Aminotransferase Levels. J. Clin. Endocrinol. Metab..

[4] National Guideline Center (2016). National Institute for Health and Care Excellence: Guidelines. Non-Alcoholic Fatty Liver Disease: Assessment and Management.

[5] Maurice J., Manousou P. (2018). Non-alcoholic fatty liver disease. Clin. Med..

[6] Shipovskaya A.A., Dudanova O.P. (2018). Intrahepatic cholestasis in nonalcoholic fatty liver disease. Ther. Arkh..

[7] Sorrentino P., Tarantino G., Perrella A., Micheli P., Perrella O., Conca P. (2005). A clinical-morphological study on cholestatic presentation of nonalcoholic fatty liver disease. Dig. Dis. Sci..

[8] Gadd V.L., Skoien R., Powell E.E., Fagan K.J., Winterford C., Horsfall L., Irvine K., Clouston A.D. (2014). The portal inflammatory infiltrate and ductular reaction in human nonalcoholic fatty liver disease. Hepatology.

[9] Jüngst C., Berg T., Cheng J., Green R.M., Jia J., Mason A.L., Lammert F. (2013). Intrahepatic cholestasis in common chronic liver diseases. Eur. J. Clin. Investig..

[10] DeLeve L.D., Kaplowitz N. (1995). Mechanisms of drug-induced liver disease. Gastroenterol. Clin. North Am..

[11] Kwo P.Y., Cohen S.M., Lim J.K. (2017). ACG Clinical Guideline: Evaluation of Abnormal Liver Chemistries. Am. J. Gastroenterol..

[12] Shirin D., Peleg N., Sneh-Arbib O., Cohen-Naftaly M., Braun M., Shochat T., Issachar A., Shlomai A. (2019). The Pattern of Elevated Liver Function Tests in Nonalcoholic Fatty Liver Disease Predicts Fibrosis Stage and Metabolic-Associated Comorbidities. Dig. Dis..

[13] Pennisi G., Pipitone R.M., Cabibi D., Enea M., Romero-Gomez M., Viganò M., Bugianesi E., Wong V.W., Fracanzani A.L., Sebastiani G. (2022). A cholestatic pattern predicts major liver-related outcomes in patients with non-alcoholic fatty liver disease. Liver Int..

[14] Moll R., Franke W.W., Schiller D.L., Geiger B., Krepler R. (1982). The catalog of human cytokeratins: Patterns of expression in normal epithelia, tumors and cultured cells. Cell.

[15] Chu P.G., Weiss L.M. (2002). Keratin expression in human tissues and neoplasms. Histopathology.

[16] Bateman A.C., Hübscher S.G. (2010). Cytokeratin expression as an aid to diagnosis in medical liver biopsies. Histopathology.

[17] Matsukuma S., Takeo H., Kono T., Nagata Y., Sato K. (2012). Aberrant cytokeratin 7 expression of centrilobular hepatocytes: A clinicopathological study. Histopathology.

[18] Schnell U., Cirulli V., Giepmans B.N. (2013). EpCAM: Structure and function in health and disease. Biochim. Biophys. Acta.

[19] Herlyn M., Steplewski Z., Herlyn D., Koprowski H. (1979). Colorectal carcinoma-specific antigen: Detection by means of monoclonal antibodies. Proc. Natl. Acad. Sci. USA.

[20] Pavšič M., Gunčar G., Djinović-Carugo K., Lenarčič B. (2014). Crystal structure and its bearing towards an understanding of key biological functions of EpCAM. Nat. Commun..

[21] Strnad J., Hamilton A.E., Beavers L.S., Gamboa G.C., Apelgren L.D., Taber L.D., Sportsman J.R., Bumol T.F., Sharp J.D., Gadski R.A. (1989). Molecular cloning and characterization of a human adenocarcinoma/epithelial cell surface antigen complementary DNA. Cancer Res..

[22] Litvinov S.V., Bakker H.A., Gourevitch M.M., Velders M.P., Warnaar S.O. (1994). Evidence for a role of the epithelial glycoprotein 40 (Ep-CAM) in epithelial cell-cell adhesion. Cell Adhes. Commun..

[23] de Boer C.J., van Krieken J.H., Janssen-van Rhijn C.M., Litvinov S.V. (1999). Expression of Ep-CAM in normal, regenerating, metaplastic, and neoplastic liver. J. Pathol..

[24] Yoon S.M., Gerasimidou D., Kuwahara R., Hytiroglou P., Yoo J.E., Park Y.N., Theise N.D. (2011). Epithelial cell adhesion molecule (EpCAM) marks hepatocytes newly derived from stem/progenitor cells in humans. Hepatology.

[25] Safarikia S., Carpino G., Overi D., Cardinale V., Venere R., Franchitto A., Onori P., Alvaro D., Gaudio E. (2020). Distinct EpCAM-Positive Stem Cell Niches Are Engaged in Chronic and Neoplastic Liver Diseases. Front. Med..

[26] Kleiner D.E., Brunt E.M., Van Natta M., Behling C., Contos M.J., Cummings O.W., Ferrell L.D., Liu Y.C., Torbenson M.S., Unalp-Arida A. (2005). Design and validation of a histological scoring system for nonalcoholic fatty liver disease. Hepatology.

[27] Heinemann F., Birk G., Stierstorfer B. (2019). Deep learning enables pathologist-like scoring of NASH models. Sci. Rep..

[28] Heinemann F., Gross P., Zeveleva S., Qian H.S., Hill J., Höfer A., Jonigk D., Diehl A.M., Abdelmalek M., Lenter M.C. (2022). Deep learning-based quantification of NAFLD/NASH progression in human liver biopsies. Sci. Rep..

[29] Shirin D., Tobar A., Bendersky A.G., Velders M.P., Harif Y., Naamneh R., Shlomai A. Liver test-derived R factor is associated with portal hypertension in patients with non-alcoholic fatty liver disease. Proceedings of the Easl ILC.

[30] Desmet V.J. (2011). Ductal plates in hepatic ductular reactions. Hypothesis and implications. I. Types of ductular reaction reconsidered. Virchows Arch..

[31] Onofrio F.Q., Hirschfield G.M. (2020). The Pathophysiology of Cholestasis and Its Relevance to Clinical Practice. Clin. Liver Dis..

[32] Desmet V.J. (2011). Ductal plates in hepatic ductular reactions. Hypothesis and implications. II. Ontogenic liver growth in childhood. Virchows Arch..

[33] Desmet V.J. (2011). Ductal plates in hepatic ductular reactions. Hypothesis and implications. III. Implications for liver pathology. Virchows Arch..

[34] Manco R., Clerbaux L.A., Verhulst S., Bou Nader M., Sempoux C., Ambroise J., Bearzatto B., Gala J.L., Horsmans Y., van Grunsven L. (2019). Reactive cholangiocytes differentiate into proliferative hepatocytes with efficient DNA repair in mice with chronic liver injury. J. Hepatol..

[35] Sato K., Marzioni M., Meng F., Francis H., Glaser S., Alpini G. (2019). Ductular Reaction in Liver Diseases: Pathological Mechanisms and Translational Significances. Hepatology.

[36] Carpino G., Cardinale V., Folseraas T., Overi D., Floreani A., Franchitto A., Onori P., Cazzagon N., Berloco P.B., Karlsen T.H. (2018). Hepatic Stem/Progenitor Cell Activation Differs between Primary Sclerosing and Primary Biliary Cholangitis. Am. J. Pathol..

[37] Weber S., Allgeier J., Denk G., Gerbes A.L. (2022). Marked Increase of Gamma-Glutamyltransferase as an Indicator of Drug-Induced Liver Injury in Patients without Conventional Diagnostic Criteria of Acute Liver Injury. Visc. Med..

[38] Irie M., Suzuki N., Sohda T., Anan A., Iwata K., Takeyama Y., Watanabe H., Fischer P., Scherberich J.E., Sakisaka S. (2007). Hepatic expression of gamma-glutamyltranspeptidase in the human liver of patients with alcoholic liver disease. Hepatol. Res..

[39] Bulle F., Mavier P., Zafrani E.S., Preaux A.M., Lescs M.C., Siegrist S., Dhumeaux D., Guellaën G. (1990). Mechanism of gamma-glutamyl transpeptidase release in serum during intrahepatic and extrahepatic cholestasis in the rat: A histochemical, biochemical and molecular approach. Hepatology.

[40] Trauner M., Fuchs C.D. (2022). Novel therapeutic targets for cholestatic and fatty liver disease. Gut.

[41] Marchianò S., Biagioli M., Morretta E., Di Giorgio C., Roselli R., Bordoni M., Bellini R., Urbani G., Massa C., Monti M.C. (2023). Combinatorial therapy with BAR502 and UDCA resets FXR and GPBAR1 signaling and reverses liver histopathology in a model of NASH. Sci. Rep..

[42] Xiang Z., Chen Y.P., Ma K.F., Ye Y.F., Zheng L., Yang Y.D., Li Y.M., Jin X. (2013). The role of ursodeoxycholic acid in non-alcoholic steatohepatitis: A systematic review. BMC Gastroenterol..

